# Fuzzy Decision-Based Efficient Task Offloading Management Scheme in Multi-Tier MEC-Enabled Networks

**DOI:** 10.3390/s21041484

**Published:** 2021-02-20

**Authors:** Md Delowar Hossain, Tangina Sultana, Md Alamgir Hossain, Md Imtiaz Hossain, Luan N. T. Huynh, Junyoung Park, Eui-Nam Huh

**Affiliations:** Department of Computer Science and Engineering, Kyung Hee University, Global Campus, Yongin-si 17104, Korea; delowar@khu.ac.kr (M.D.H.); tangina@khu.ac.kr (T.S.); alamgir@khu.ac.kr (M.A.H.); hossain.imtiaz@khu.ac.kr (M.I.H.); luanhnt@khu.ac.kr (L.N.T.H.); parkhans@khu.ac.kr (J.P.)

**Keywords:** multi-access edge computing, orchestrator, task offloading, fuzzy logic, 5G

## Abstract

Multi-access edge computing (MEC) is a new leading technology for meeting the demands of key performance indicators (KPIs) in 5G networks. However, in a rapidly changing dynamic environment, it is hard to find the optimal target server for processing offloaded tasks because we do not know the end users’ demands in advance. Therefore, quality of service (QoS) deteriorates because of increasing task failures and long execution latency from congestion. To reduce latency and avoid task failures from resource-constrained edge servers, vertical offloading between mobile devices with local-edge collaboration or with local edge-remote cloud collaboration have been proposed in previous studies. However, they ignored the nearby edge server in the same tier that has excess computing resources. Therefore, this paper introduces a fuzzy decision-based cloud-MEC collaborative task offloading management system called FTOM, which takes advantage of powerful remote cloud-computing capabilities and utilizes neighboring edge servers. The main objective of the FTOM scheme is to select the optimal target node for task offloading based on server capacity, latency sensitivity, and the network’s condition. Our proposed scheme can make dynamic decisions where local or nearby MEC servers are preferred for offloading delay-sensitive tasks, and delay-tolerant high resource-demand tasks are offloaded to a remote cloud server. Simulation results affirm that our proposed FTOM scheme significantly improves the rate of successfully executing offloaded tasks by approximately 68.5%, and reduces task completion time by 66.6%, when compared with a local edge offloading (LEO) scheme. The improved and reduced rates are 32.4% and 61.5%, respectively, when compared with a two-tier edge orchestration-based offloading (TTEO) scheme. They are 8.9% and 47.9%, respectively, when compared with a fuzzy orchestration-based load balancing (FOLB) scheme, approximately 3.2% and 49.8%, respectively, when compared with a fuzzy workload orchestration-based task offloading (WOTO) scheme, and approximately 38.6%% and 55%, respectively, when compared with a fuzzy edge-orchestration based collaborative task offloading (FCTO) scheme.

## 1. Introduction

Nowadays, with the rapid evolution of communication technology and the enormous popularity of high-demand applications (e.g., the Internet of vehicles, mobile augmented reality, map navigation, face/fingerprint/iris recognition, mobile healthcare, web browsing, cloud gaming, image identification), a huge number of devices are attached to the Internet of Things (IoT) infrastructure [1,2,3,4,5]. In conventional networking infrastructures, the demand poses an enormous burden due to the generation of huge volumes of data from using these devices. Moreover, storage capacity and computing capabilities in user devices is restricted. Due to these constraints, user devices cannot handle massive numbers of tasks, and it affects both quality of service (QoS) and performance. Therefore, these devices tend to offload their tasks to more powerful computing devices [6]. To resolve the above limitations, the mobile cloud computing (MCC) approach was introduced [7]. Thus, the workload of user devices and the processing latency are significantly reduced from offloading computation tasks to the MCC server. However, the location of the MCC server is on the core network, far from the user devices. Therefore, when a user wants to offload a task to the MCC server, the data must travel through the entire access network. The same scenario has to be followed when the processed results return. As a result, the MCC-based approach suffers from high transmission delays, data leakage, and compromised privacy due to the long-distance routing [8]. To reduce this network latency is very difficult if using the existing infrastructure. Therefore, for applications that need low latency in a real-time service environment, the MCC-based solution is not suitable. To cope with these challenges, ETSI proposed in December 2014 an emerging technology named mobile edge computing. In September 2017, ETSI removed the word mobile from multi-access edge computing (MEC) and officially renamed it multi-access edge computing [9,10]. MEC is an innovative network paradigm that brings the storage and computing resources to the network edge. As a result, it can overcome long transmission latency and the deficiencies from network congestion in the MCC system. Since the location of MEC servers is very close to the user terminals, end-to-end latency between the edge server and user device is significantly shortened. Therefore, the user can receive feedback immediately after processing, and this significantly improves QoS. Table 1 compares MCC and MEC [11,12].

MEC is one of the premier ideas for rapidly computing user tasks offloaded to the edge server. The advantage of this technology is that users get the needed computing resources with only one-hop wireless transmission. Compared to MCC, it does not need to go through the core network to transmit the task to MEC servers. This reduces the delay and satisfies the low-latency requirements of different applications. In addition, the task’s processed results return directly from the MEC server, which can alleviate the risk to privacy and helps to protect sensitive data. Moreover, the edge server (as well as user devices) can themselves collaboratively process the service workloads. As a result, it can save bandwidth, because most of the task is processed locally by the user device and the edge server, without sending the task to the cloud. Therefore, to handle context-aware and latency-sensitive applications, some researchers have proposed a framework for collaboration between the edge server and user devices to complete computed tasks [13]. Despite the multi-dimensional benefits of MEC, it faces challenges owing to finite storage capacity and limited computation resources. With the increase in high-demand applications and the popularity of smart mobile devices, the distinct edge server cannot efficiently handle multiple offload requests. To utilize adequate computing resources of remote cloud servers and benefit from using a MEC server, a collaborative cloud-MEC-based task offloading approach was proposed recently [14,15]. In collaborative offloading, there are still some challenges, such as how to decide where to offload the task (to either a MEC server or a cloud server). Therefore, the collaborative approach is more complicated in a dynamic environment. To exploit the advantages of unlimited storage space and powerful computing capabilities of a cloud server, and to utilize nearby MEC servers, a collaborative cloud-MEC-based FTOM scheme is introduced in this study. The novelty of our work is to improve the rate of successfully executing offloaded tasks and to reduce completed-task latency by utilizing the computing resources of nearby MEC servers that have excess computing resources. The key contributions of this paper are as follows:

- We investigate a low-complexity cloud-MEC-based offloading scheme to ensure QoS and accommodate more workload in the multi-tier MEC-enabled network.
- We develop a fuzzy decision-based, efficient task offloading management scheme by considering a vertical (local MEC with remote cloud) as well as horizontal (peer offloading among nearby MEC servers) task offloading scheme to meet the diverse needs of users.
- Based on the the states of server utilization, the delay sensitivity of the task, and the network conditions, the FTOM scheme can make a dynamic decision on where to offload the incoming task: local MEC, nearby MEC, or a cloud server.
- To improve resource utilization efficiency and the rate of successfully executed offloaded tasks, our system prefers to offload latency-sensitive tasks to local or neighboring MEC servers, whereas delay-tolerant, high resource-demand tasks go to a remote server.
- Performance evaluation demonstrates the effectiveness of our proposed FTOM scheme, compared to its competitors, for three different types of application: infotainment (I), augmented reality (AR), and health monitoring (HM).

The remainder of this paper is structured as follows. The related works on task offloading in the MEC-enabled networks are illustrated briefly in Section 2. Afterwards, the problem scenario and our proposed model are described in Section 3. Our introduced FTOM scheme for efficient task offloading management is presented in Section 4. Performance evaluations are illustrated in Section 5 and results summary of different evaluation metrics are presented in Section 6. The paper is finally concluded and future research suggested in Section 7.

## 2. Related Work

Task offloading and allocation of resources are primary key points of MEC-enabled networks. Based on previous research, these are divided into three main categories: binary or full offloading (the task cannot partition during processing) [16,17,18], partial offloading (the task is decomposed into several parts at the same time for local computing or for offloading) [19,20,21], and collaborative task offloading (integration between the edge and the cloud) [22,23,24,25]. For binary computation offloading (BCO), the tasks can be processed by the user devices themselves or by offloading them to the nearest edge servers. This scheme is mostly an NP-hard problem. To solve the problem of having multi-user participation and restrictive objectives, game theory is extensively used. Bi and Zhang [16] proposed a BCO policy to process a task with either user devices or by offloading it to an edge server for a multi-user MEC system. By using wireless power transfer (WPT), the users are wirelessly powered from the base station. Wang et al. [17] proposed a three-layer traffic system based on queueing theory for moving vehicle-based edge nodes to minimize the offloaded response time. Messous et al. [18] introduced a game theory-based strategy for solving offloaded problems with heavy task computations in unmanned aerial vehicles (UAVs). Recently, partial computational offloading (PCO) has gained widespread attention from many researchers into MEC-enabled networks. In this offloading model, a task is partitioned into some parts that are executed locally by mobile devices and other parts that are offloaded to, and processed by, MEC servers. The authors of [19] proposed a PCO approach for a single-user MEC system to minimize energy consumption and the task execution latency based on Lagrangian dual decomposition. To solve single-user PCO problems in latency-constrained networks, Ning et al. [20] apply a branch-and-bound algorithm. On the other hand, for multi-user PCO problems, a heuristic iterative algorithm was proposed for making offloading decisions and allocation of resources dynamically. To reduce latency for all user devices, Ren et al. [21] proposed a strategy named optimal closed-form data segmentation in partial computation offloading schemes for time-division multiple access based multi-user MEC systems.

However, due to the resource restrictions and limited storage capacity of the MEC server, researchers have proposed cloud-MEC-based collaborative integration to reap the benefits of both technologies. Most of the previous researchers proposed two-tier cloud-MEC-based vertical offloading [22,23,24,25] and ignored horizontal offloading among nearby MEC servers in the same tier. Deng et al. [22] introduced a cloud-edge computing system to reduce power consumption as well as delay by formulating a mixed-integer nonlinear programming (MINLP) problem. By adopting the fiber-wireless (FiWi) access network, Guo and Liu [23] proposed a collaborative cloud-MEC-based task offloading scheme. To obtain better offloading performance, a game-theory-based algorithm was used. To reduce the cost of the capacity, Lin et al. [24] constructed a three-tier cloud-edge system by using an iterative optimization algorithm. To minimize the network transmission load, Huang et al. [25] introduced a service orchestration scheme based on software-defined networking (SDN) technology. Furthermore, a heuristic algorithm was adopted to make the offloading decision between the cloud and the edge system. On the other hand, to utilize nearby MEC servers, some researchers focused on horizontal offloading between local MEC and nearby MEC servers in the same tier. To minimize the transmission distance and increase the capacity of edge caching systems, Yuan et al. [26] proposed a cooperation approach among edge clouds. Hossain et al. [27] used a collaboration approach based on fuzzy logic among MEC servers to reduce the task failure rate and service time. Moreover, Fan et al. [28] used a cooperation approach between different servers for balancing the computation workload.

Generally, the edge computing environment is dynamic and uncertain. On the other hand, fuzzy logic is one of the best-employed methods for rapidly changing uncertain systems. Therefore, for efficient task offloading management, the FTOM scheme is proposed. The main advantages of using fuzzy logic are that its complexity is low, compared with other decision-making algorithms [29,30,31], and it is significantly applied to workload management, vehicle routing, task scheduling, and network congestion-mitigation problems [32,33,34]. To satisfy the various security requirements in real time for mobile users, Li et al. [35] introduced a security service-chaining approach based on fuzzy logic for mobile edge computing. Nguyen et al. [36] proposed a fuzzy decision-based flexible task-offloading scheme for IoT applications. To minimize latency and the task failure rate, a fuzzy-based mobile edge orchestrator policy is used as a controller for application placement. Soleymani et al. [37] used fuzzy logic for the trust management system in a VANET. The proposed trust model executes a sequence of security checks to ensure vehicles are authorized. On the other hand, to determine the target server for task offloading, Sonmez et al. [38] used two stages of fuzzy operation. The best-candidate edge server is found in the first stage from among all the edge servers. The target server is selected by comparing the candidate edge server with the cloud in the second stage. Our proposed system, however, uses only a single stage of fuzzy logic operation to select the optimal target server. In [27], Hossain et al. considered a collaborative approach for task offloading based on fuzzy logic. In this paper, authors considered local MEC and neighboring MEC servers to select the target server for task offloading decisions. To calculate the center of gravity (COG) value for choosing the target server to offload the task, authors did not consider the remote cloud server. Moreover, for performance evaluation, authors considered latency-sensitive AR application. However, for offloading tasks, our proposed FTOM scheme selects the optimal server from among local MEC, nearby MEC, or remote cloud servers. That is why we have considered a new input variable, named WAN bandwidth. The important role of WAN bandwidth is in making the decision about offloading the task to the remote cloud or not. Moreover, in FTOM scheme, for performance evaluation, we have considered latency-sensitive AR and HM applications and delay-tolerant infotainment application. The two key activities of the FTOM scheme are monitoring the continuously changing network conditions and finding the optimal target server for task offloading. As far as we know, an FTOM scheme for MEC-enabled networks has not been evaluated yet in this domain.

## 3. Problem Scenario and System Model

### 3.1. Problem Scenario

**Figure 1:**
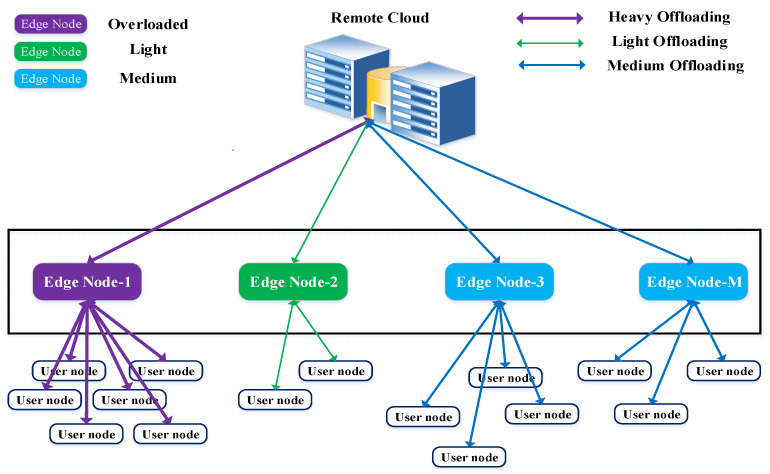
The overloaded problem in a multi-user MEC network.

**Figure 2:**
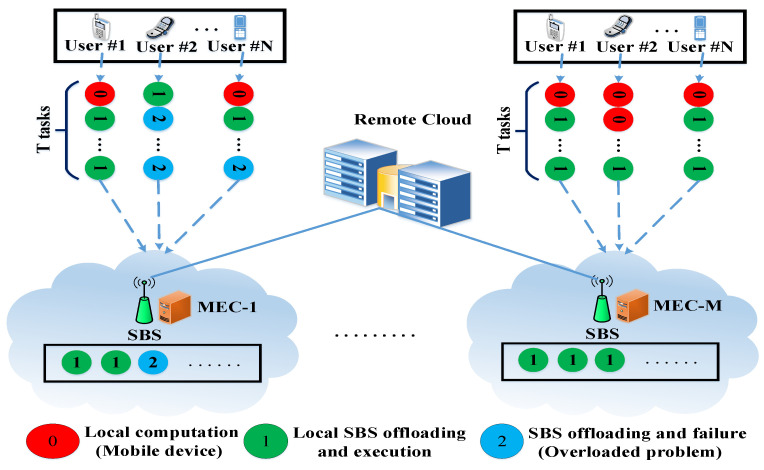
Multi-server multi-user MEC network.

In MEC-enabled networks, task offloading is one of the challenging issues because of the delay constraint and limited computing resources. Moreover, congestion is caused from offloading multiple tasks from various users to the same edge server. Therefore, many users’ processing tasks on the MEC server are left waiting in the queue. As a result, the processing delay is longer for all tasks because of the overload. Figure 1 and Figure 2 show such scenarios, where some edge nodes are lightly loaded and some nodes are overloaded from too many user requests. Therefore, it is not always a better decision to offload a computing task to the closest edge server. From Figure 1, we can see that edge node-1 is already overloaded due to heavy user requests. In this situation, the overload tasks are forwarded to the remote cloud for processing. However, the nearby edge node-2 is lightly loaded and has more resources available to process computing tasks. This node can undoubtedly overcome the overload problem for edge node-1 without sending tasks to the remote cloud. In the ongoing 5G network, multiple edge servers are deployed near user devices within range of mobile communication. Therefore, users have multiple options for offloading tasks to nearby edge servers in order to receive services. On the other hand, when there are multiple edge servers available in MEC networks, it becomes a challenging issue to decide which edge server is best for task offloading. Thus, the design of an efficient task offloading mechanism is important, because QoS varies based on the task offloading decisions. Figure 1 shows the following two significant challenges faced when offloading tasks in MEC networks:

1. Should the edge server or the remote server be used to offload the computing task?
2. Which edge server is preferred for offloading the task?

To clearly understand the offloading problem, Figure 2 shows a multi-user MEC network scenario in detail. This network consists of M={1,2,3,…,M} small base stations (SBSs), and a single MEC server is deployed in each SBS. There are N={1,2,3,…,N} user devices and T={1,2,3,…,T} independent tasks from each user. We denote the computing capacity of the edge server as $r^{mec}$, and this server receives its mobile workload from *N* users, $\phi_{1},\phi_{2},\ldots ,\phi_{n}$. Based on the user device capacity, some tasks are executed locally by the device, and the rest of the tasks are offloaded to a local MEC server. If the received workload exceeds the capacity of the edge server (i.e., $\sum \phi >r^{mec}$), it is hard to execute another task on this server. Therefore, due to the excessive workload, task 2 fails, as shown in Figure 2. To explore the neighboring SBSs and the remote cloud, we observed the following:

1. To overcome the local MEC server overload problem and utilize the neighboring MEC servers with the remote cloud, we can add a orchestrator management layer for efficient task offloading among MEC servers within the cloud.
2. Based on the task size, network condition, and delay sensitivity of the task, we can decide whether task offloading is more efficient if done by a local MEC server, a neighboring MEC server, or the remote cloud.
3. The rate of successfully executed tasks can improve, and task completion time can be significantly reduced, by offloading the task collaboratively among the MEC servers and the remote cloud server.

### 3.2. The Role of an Orchestrator Management Scheme

**Figure 3:**
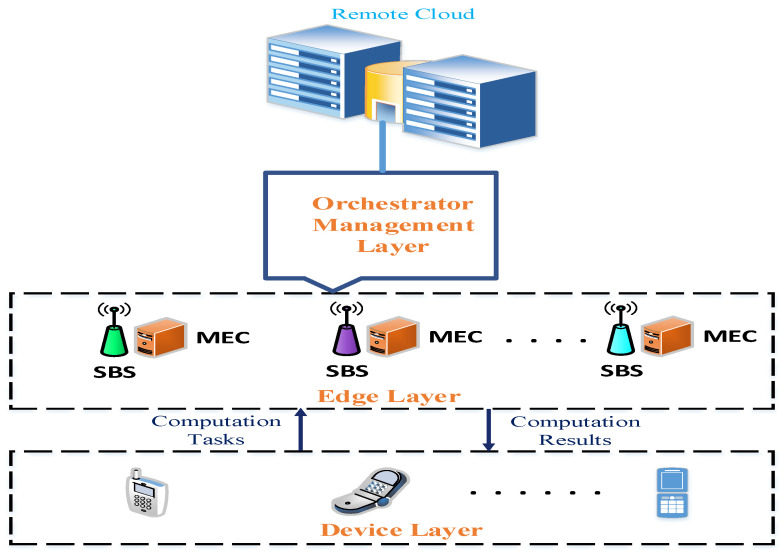
Orchestrator management scheme.

To solve the overload problem in a distinct edge sever, we include a management layer for task orchestration among the MEC servers and the cloud in a multi-tier MEC-enabled network, which is shown in Figure 3.

**Figure 4:**
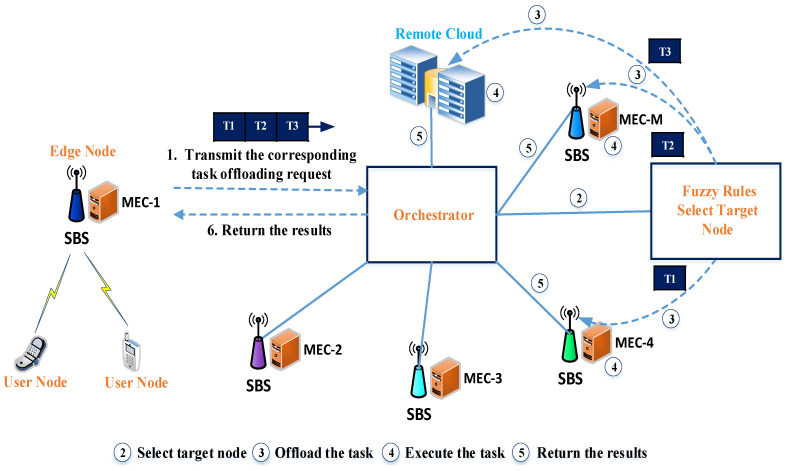
The role of the orchestrator and the flow of the task offloading process.

Without an orchestrator, all incoming user requests are offloaded and executed by the local MEC server. Therefore, it faces heavy congestion because of the numerous user requests, and sometimes, resources are not utilized efficiently. As depicted in Figure 3, we incorporate the orchestrator management layer between the edge layer and the remote cloud. Numerous devices, such as smartphones and sensing devices, are deployed on the device layer of the network and want to offload their computing tasks to an edge server or a remote server. The edge layer consists of multiple SBSs where a single MEC server is equipped at each SBS. The orchestrator management layer is responsible for collecting all information, including the computation resources of the MEC servers, the network information, and the input task sizes. Based on this information, it selects the optimal target node for task computing to ensure a sophisticated computation balance. Figure 4 describes the role of the orchestrator and the task offloading process. The user node selects the local SBS for task requesting. We assume that the task is already offloaded from the end-user device to the local edge node, and that each task is independent. There are six steps required to execute the process. (1) SBS integrates the edge node with task offloading information together and transmits the corresponding task offloading request to the orchestrator along with its requirements. (2) The orchestrator acts as the decision-maker of the system and, based on fuzzy rules, it decides where (i.e., in which resource) the tasks will be executed. When an edge node is connected to the network, the orchestrator links that node to the system. Then, during the offloading process, the orchestrator finds the best offload destination (i.e., the node that will execute the offloaded task) in the system. (3) The system sends the task to the optimal edge node based on fuzzy rules. (4) The selected edge node executes the task. (5) After executing the task, the result is returned to the orchestrator. (6) The orchestrator forwards the result to the corresponding edge node.

### 3.3. System Model

**Figure 5:**
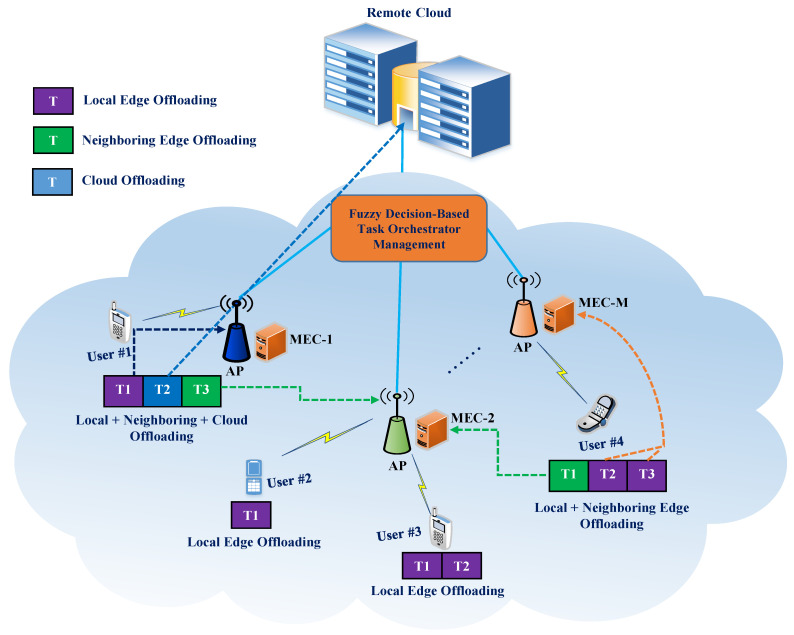
Proposed multi-tier MEC system architecture.

The proposed model is an integration framework with one centralized cloud server, *M* access points (AP), and many user devices which are all shown in Figure 5. There is a single MEC server in each AP which has limited storage and computing resources for processing tasks. The combination of the AP and its associated MEC server is considered an edge node. On the other hand, a centralized cloud server has a huge amount of storage capacity and powerful computing resources. Mobile users utilize wireless local area network to access edge resources, whereas wide area network connections are used if devices offload their tasks to a remote cloud server. We assume there are *N* user devices (UDs) where each user has *T* independent tasks. We denote the set of UDs as $U,U=\{U_{i}|i=1,2,3,\ldots ,N\},|U|=N$, and the set of tasks that need to be executed for each user in the network is $T=\{T_{i}|i=1,2,3,\ldots ,T\}$. Each computation task is described by the following: $T_{i}=\left\{\tau_{i},\psi_{i},d_{max}^{i}\right\}$. For task $T_{i}$, $\tau_{i}$ denotes the size of the task that needs to be offloaded for computation; $\psi_{i}$ represents the required CPU cycle for task processing, which varies for various applications; and $d_{max}^{i}$ indicates the maximum tolerable latency of $T_{i}$. Moreover, we define the set of servers as M={1,2,3,…,M,M+1}, where $\left\{MEC_{i}|i=1,...M\right\}$ denotes the MEC servers and server M+1 represents the remote cloud server. For each MEC server, $MEC_{i}=\left\{r_{max}^{i},s_{max}^{i}\right\}$, where $r_{max}^{i}$ is the maximum resource capability of $MEC_{i}$, and $s_{max}^{i}$ is the local storage capacity of $MEC_{i}$. We assume that each $MEC_{i}$ server has one host that operates four VMs. The resource capacity of each VM is 10 GIPS. If the required amount of resources is less than or equal to $r_{max}^{i}$, then the task will be executed only by $MEC_{i}$. On the other hand, the VMs running on the global cloud server are tens of times more powerful than the edge server in our scenario. The main aim of this study is to design an efficient cloud-MEC-based task offloading management approach to ensure satisfactory service requirements and reduce the overall latency.

For each task, we consider the task offloading decision among the MEC servers and the cloud to be represented by

(1) $$O_{ntm}\in \left\{0,1\right\}$$

where the *t*-th task of user device *n* is allocated to server *m*. Here, n∈N, t∈T, m∈M. When $O_{ntm}=0$, the user device *n* will decide to offload its *t*-th task to the cloud server. Then, we have $O_{ntm}=1,\forall m\in M\setminus \left\{M+1\right\}$. In this scenario, every task must use one of those servers for processing. Mathematically, it can be represented as follows:

(2) $$\sum_{m=1}^{M+1}O_{ntm}=1$$

So, we can write the different computing modes mathematically, as given in Equation (3).

(3) $$\left\{\begin{array}{c} \sum_{m=1}^{M}O_{ntm}=1,\;MEC\;computing\\ O_{ntm+1}=1,\;Cloud\;computing \end{array}\right.$$

for any n∈N and t∈T.

In our proposed architecture, the following three cases may occur during task offloading.

1. Case 1: In this scenario, we consider the task to be offloaded and processed only by the local MEC server. For example, in Figure 5, we can see that User #2 has only one task (T1) and User #3 has two tasks (T1 and T2). Because the local MEC server has enough capacity, both User #2 and User #3 process their computing tasks fully at the local MEC server.
2. Case 2: In this scenario, the offloaded task is executed by computation peer offloading between the local and nearby MEC servers. In describing Case 2, consider User #4 as having three tasks (T1, T2, and T3). Based on our proposed FTOM scheme, tasks T2 and T3 are processed locally because of the capabilities of the local MEC server, and task T1 is processed by a nearby MEC server.
3. Case 3: In this scenario, the offloaded task is executed through collaboration among a local MEC server, a neighboring MEC server, and the remote cloud. User #1 describes Case 3 and has three tasks (T1, T2, and T3). Based on the task orchestration management decision, T1 is processed by the local MEC server, T2 is handled by the remote cloud server, and T3 is executed by the nearby MEC server.

## 4. Fuzzy Decision-Based Task Offloading Management

**Figure 6:**
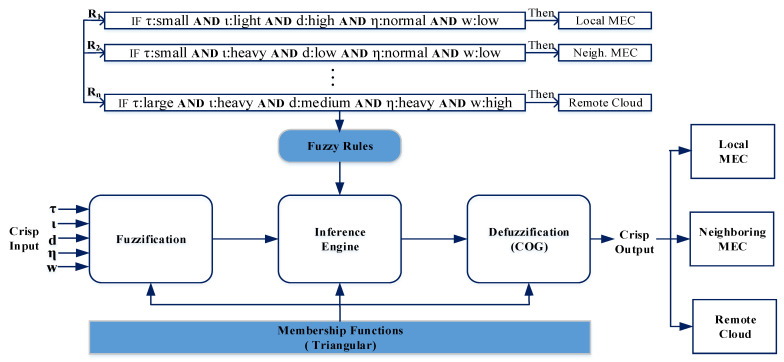
The proposed fuzzy logic architecture.

For the efficient task offloading management of multi-tier MEC-enabled networks, we propose a fuzzy decision-based scheme for a multitude of reasons. The environment of edge computing is dynamic, and the stages of resources continuously change based on the offload requests. Due to this uncertainty, it is difficult to make a decision as to where a task should execute because we do not know the number of incoming user requests in advance. Moreover, task offloading management is basically online and considered an NP-hard problem. Therefore, we cannot apply conventional offline optimization techniques [36,38]. To handle these unpredictable environments, we need a low-complexity problem-solving technique. In addition, there are many input and output parameters involved in the MEC-enabled network environment, and these parameters are a part of the environmental behavior. This approach is inherently fuzzy. In this respect, fuzzy logic is one of the best alternatives to deal with the above-mentioned rapidly changing uncertain system. The advantage of fuzzy logic is that its complexity is very low, which is basically a very important criterion for an online algorithm [30]. Figure 6 shows the fuzzy logic architecture used in our proposed model. The main objective of our proposed fuzzy decision-based scheme is to identify a target server for the offloaded task by monitoring different factors, including the incoming task’s size, the network’s condition, and the resources already utilized in the servers. The three main steps of the fuzzy reasoning mechanism are described as follows.

### 4.1. Fuzzification

During fuzzification, a crisp value is transformed into a fuzzy value by using membership functions (MFs). The crisp set of input parameters, which are described in Table 2, is the input for the fuzzy logic engine. It basically determines the degree of input data having the appropriate fuzzy sets by using the MFs. For efficient task offloading management, we define five significant fuzzy input variables: task size, local MEC VM utilization, network delay, neighboring MEC VM utilization, and WAN bandwidth. We represent these input variables mathematically as follows:

(4) Ω=[τ,ι,d,η,w]

where τ indicates the length of the incoming task in order to determine the task execution time; ι and η, respectively, represent the status of local MEC server and neighboring MEC server computational resources; *d* denotes the network delay; and *w* represents the WAN bandwidth. If the local MEC server is heavily congested and the latency of the network is very low, it will be advantageous to compute the incoming task by the neighboring MEC server. On the other hand, due to the heavily loaded neighboring MEC server and high network delay for handling large incoming requests, it is better to process the task in a local MEC server. The role of *w* is in making the decision about offloading the task to the remote cloud or not. If the local and neighboring servers are heavily loaded and WAN bandwidth is high, then it is appropriate to execute the incoming task to the remote cloud.

**Figure 7:**
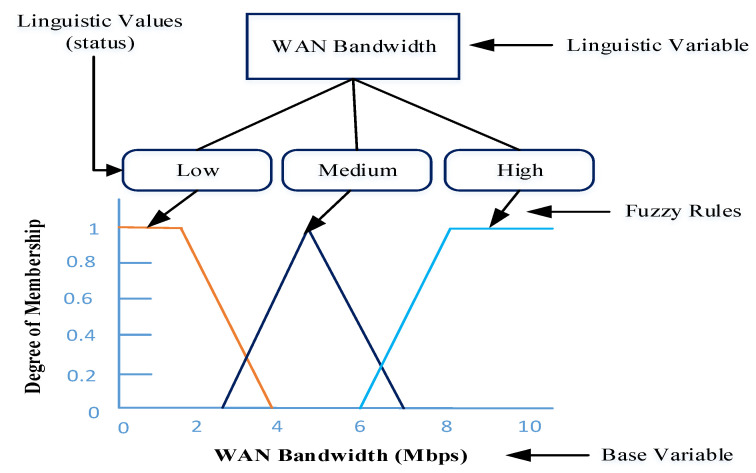
Example of linguistic variables for WAN bandwidth.

Generally, a fuzzy logic system (FLS) uses non-numerical linguistic variables, such as Small, Medium, and Heavy, which come from natural language. Our FTOM scheme uses different linguistic variables to indicate the input parameters. Every base variable is represented by a linguistic variable, where the values are real numbers within a specific range. On the other hand, a linguistic variable is defined by using different terms that are the approximate value of a base variable. In Figure 7, we use a linguistic variable to represent WAN bandwidth. Based on the different bandwidths, the linguistic values for WAN bandwidth are Low, Medium, and High. For example, when the WAN bandwidth is up to 4 Mbps, we consider the bandwidth to be low. Moreover, we consider WAN bandwidth to be medium when the bandwidth range is between 3 Mbps and 7 Mbps. Furthermore, if the bandwidth range is between 6 Mbps and 21 Mbps, we consider the bandwidth to be high. A linguistic variable can be defined by using triplets ($V,R,\Omega_{V}$), where *V* represents a fuzzy input variable such as network delay or WAN bandwidth, *R* denotes range of the variable, and $\Omega_{V}$ defines the set of linguistic terms for the fuzzy variable [39]. A linguistic variable for WAN bandwidth can be represented, based on Figure 7, as follows:

(5) $$Linguistic\;variable,w=\left\{\begin{array}{c} w=WAN\;Bandwidth\\ R={ℜ}^{+}\\ \Omega_{w}=\left(Low,Medium,High\right) \end{array}\right.$$

In this paper, we use three different linguistic terms such as Small (S), Medium (M), and Large (L) to represent the linguistic variable having task size τ. For network delay *d* and WAN bandwidth *w*, we use the linguistic terms Low (L), Medium (M), and High (H). Furthermore, the other two linguistic variables, ι and η, are Light (L), Normal (N), and Heavy (H). Mathematically, each of the above-mentioned input linguistic variables and their different terms are represented as follows:

(6) $$\left\{\begin{array}{c} \Omega_{\tau }\left(x\right)=\left[\mu_{\tau }^{S}\left(x\right),\;\mu_{\tau }^{M}\left(x\right),\;\mu_{\tau }^{L}\left(x\right)\right]\;\mathrm{and}\\ \Omega_{i}\left(x\right)=\left[\mu_{i}^{L}\left(x\right),\;\mu_{i}^{M}\left(x\right),\;\mu_{i}^{H}\left(x\right)\right],\;\mathrm{where}\;j\in \left\{d,w\right\},\mathrm{and}\\ \Omega_{j}\left(x\right)=\left[\mu_{j}^{L}\left(x\right),\;\mu_{j}^{N}\left(x\right),\;\mu_{j}^{H}\left(x\right)\right],\;\mathrm{where}\;i\in \left\{\iota ,\eta \right\} \end{array}\right.$$

#### Membership Functions

MFs play an important role in the performance of FLS. We use MFs for mapping the input variables to a membership value. It returns a value in the range [0, 1], which indicates the membership degree. For each fuzzy variable, we define a set of MFs. Mathematically, it can be characterized by using Equation (7).

(7) $$A_{Fuzzy}=\left\{\left(x,\;\mu_{A}\left(x\right)\right):x\in X,\mu_{A}\left(x\right)\in \left[0,1\right]\right\}$$

Here, $\mu_{A}\left(x\right)$ represents the membership function of A. It quantifies the degree to which x belongs to A. The range of membership values is from 0 to 1, i.e., $\mu_{A}\left(x\right)\in \left[0,1\right]$, where *x* represents the element in a fuzzy set. According to Ω in Equation (4), we have used five fuzzy input variables. Based on the fuzzy input variable, we use five MF sets, and each set includes three different linguistic terms, which are used in the fuzzification steps. The MFs are represented in various forms such as Gaussian, sigmoid, singleton, trapezoidal, or triangular [40]. In this paper, we use the triangular MF form because of its low complexity. Mathematically, the triangular MF is represented in Equation (8), where *A* is the fuzzy set. The parameters *m* and *n* indicate the lower limit and upper limit, respectively; and *p* represents the modal value of the triangle:    

(8) $$\mu_{A}^{triangular}\left(x\right)=\left\{\begin{array}{cc} 0\;; & if\;x⩽m\\ \frac{x-m}{p-m}\;; & if\;m⩽x⩽p\\ \frac{n-x}{n-p}\;; & if\;p⩽x⩽n\\ 0\;; & if\;x\geq n \end{array}\right.$$

**Figure 8:**
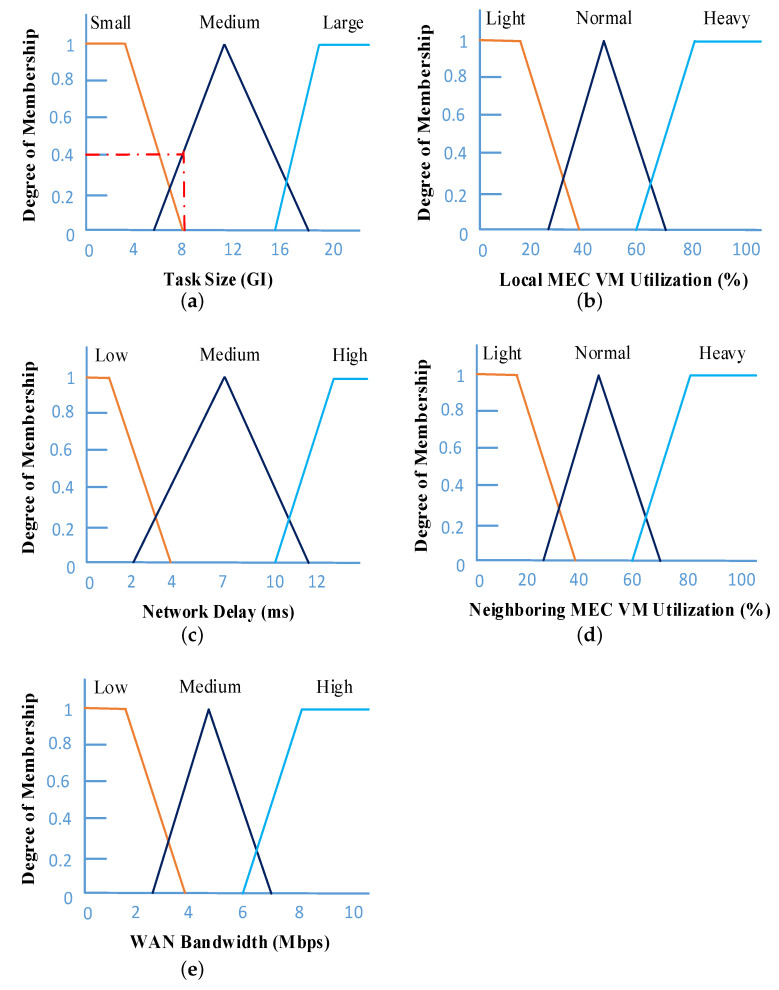
Membership functions (MFs) for the fuzzy input variables: (**a**) task size; (**b**) local MEC VM utilization; (**c**) network delay; (**d**) neighboring MEC VM utilization; (**e**) WAN bandwidth.

Determining the values used in the membership functions is critical because it has a notable impact on the overall FLS performance. Similar to other existing studies, the degree of membership values and the range of the values for each fuzzy variables are used from [31,36,38,39] because of their novel contribution to the edge computing environment based on fuzzy. The representations of MFs for the above-mentioned input fuzzy variables are shown in Figure 8. For example, if the size of the task is 8 GI, the degrees of the MF value are zero for Small, 0.4 for Medium, and zero for Large. So $\Omega_{\tau }\left(8\right)=\left[0,0.4,0\right]$, which is shown in Figure 8a.

### 4.2. Fuzzy Inference Engine

It is the process of mapping the values of the given fuzzy input variables to an output using fuzzy logic. This step is the most crucial part of the FLS. For fuzzy inference inputs, different fuzzy sets (e.g., “Small”, “Medium”, “Large”) have been considered as a confidence value. After evaluating and combining fuzzy rules, the output is generated. A fuzzy rule is constructed by a series of simple IF-THEN rules and each rule defines a fuzzy implication between condition and conclusion. A fuzzy rule has the following form:

(9) $$\left\{\begin{array}{c} If\;fvar_{1}\in A\;and\;fvar_{2}\in B\;,\;.\;.\;.\;,\;fvar_{n}\in N\;then\;f_{out}=OffDecision,\;\\ where\;A,\;B,\;,\;.\;.\;.\;,\;N\;are\;fuzzy\;sets,and\\ OffDecision\in \{localMEC,\;neighboringMEC,\;remotecloud\} \end{array}\right.$$

For fuzzification, we use five MFs sets, and we include three different linguistic terms in each set. Therefore, 243 fuzzy rules were used during the simulation. It is critical to define the fuzzy rules, because the overall performance of the system relies particularly on these rules. In this study, we use a better fuzzy rule set found empirically in [27,38]. Some examples of rules from our fuzzy rule set are given in Table 3. In each fuzzy rule, different linguistic variables are used. For example,
**IF**τ is Small**AND**ι is Light**AND***d* is High**AND**η is Normal**AND***w* is Low**THEN** offload to the local MEC server.

Basically, there are three methods (aggregation, activation, and accumulation) that are used in the inference steps [36,38]. The aggregation method (also called the rule connection method) combines multiple rules within a rule set. The activation method explains the process of applying the evaluated result of the IF part of the rule to the THEN part. Based on the fuzzy rules (Table 3) and according to Equation (6), we can calculate the fuzzy value for selecting the target server from among the local MEC, neighboring MEC, and remote cloud as follows:

(10) $$\left\{\begin{array}{c} \mu_{target}=max\left\{\mu_{localMEC}^{R1},\;\mu_{neighboringMEC}^{R2},\;\mu_{cloud}^{R3},\;.\;.\;.\;,\mu_{cloud}^{Rn}\right\},\\ where\;target\in \{localMEC,\;neighboringMEC,\;cloud\} \end{array}\right.$$

where $\mu_{localMEC}^{R1}$, $\mu_{neighboringMEC}^{R2}$, and $\mu_{cloud}^{R3}$ are represented as

(11) $$\mu_{localMEC}^{R1}=\left[\mu_{\tau }^{R1}\left(\alpha \right),\;\mu_{\iota }^{R1}\left(\beta \right),\;\mu_{d}^{R1}\left(\gamma \right),\;\mu_{\eta }^{R1}\left(\delta \right),\;\mu_{w}^{R1}\left(\theta \right)\right]$$

(12) $$\mu_{neighboringMEC}^{R2}=\left[\mu_{\tau }^{R2}\left(\alpha \right),\;\mu_{\iota }^{R2}\left(\beta \right),\;\mu_{d}^{R2}\left(\gamma \right),\;\mu_{\eta }^{R2}\left(\delta \right),\;\mu_{w}^{R2}\left(\theta \right)\right]$$

(13) $$\mu_{cloud}^{R3}=\left[\mu_{\tau }^{R3}\left(\alpha \right),\;\mu_{\iota }^{R3}\left(\beta \right),\;\mu_{d}^{R3}\left(\gamma \right),\;\mu_{\eta }^{R3}\left(\delta \right),\;\mu_{w}^{R3}\left(\theta \right)\right]$$

where α, β, γ, δ, and θ represent the value of crisp input parameters τ, ι, *d*, η, and *w* respectively, in the fuzzy inference system. We can use a simple example to describe the inference process: 7 GI, 70%, 3 ms, 35%, and 4 Mbps are the values of α, β, γ, δ, and θ respectively. For the explanation, we considered only three rules (R1, R2, and R3) from Table 3. Then, we put these values into Equations (11)–(13). During our experiment, we considered the Minimum function in the activation phase, which is the most commonly used activation function. Therefore, we applied the aggregation and activation phases to rules R1, R2, and R3 to select the target server.

(14) $$\mu_{localMEC}^{R1}=min\left[\mu_{\tau }^{R1}\left(7\right),\;\mu_{\iota }^{R1}\left(70\right),\;\mu_{d}^{R1}\left(3\right),\;\mu_{\eta }^{R1}\left(35\right),\;\mu_{w}^{R1}\left(4\right)\right]$$

(15) $$\mu_{neighboringMEC}^{R2}=min\left[\mu_{\tau }^{R2}\left(7\right),\;\mu_{\iota }^{R2}\left(70\right),\;\mu_{d}^{R2}\left(3\right),\;\mu_{\eta }^{R2}\left(35\right),\;\mu_{w}^{R2}\left(4\right)\right]$$

(16) $$\mu_{cloud}^{R3}=min\left[\mu_{\tau }^{R3}\left(7\right),\;\mu_{\iota }^{R3}\left(70\right),\;\mu_{d}^{R3}\left(3\right),\;\mu_{\eta }^{R3}\left(35\right),\;\mu_{w}^{R3}\left(4\right)\right]$$

Based on the fuzzification of input variables in Table 2, the fuzzy rules in Table 3, and MFs of the fuzzy input variables in Figure 8, we obtained fuzzy values for $\mu_{localMEC}^{R1}$, $\mu_{neighboringMEC}^{R2}$, and $\mu_{cloud}^{R3}$ are as follows:

(17) $$\mu_{localMEC}^{R1}=min\left[0.2,\;0,\;0,\;0.2,\;0\;\right]=\;0$$

(18) $$\mu_{neighboringMEC}^{R2}=min\left[0.2,\;0.5,\;0.3,\;0.2,\;0.5\;\right]=\;0.2$$

(19) $$\mu_{cloud}^{R3}=min\left[0.2,\;0.5,\;0.25,\;0,\;0\;\right]=\;0$$

Finally, to determine the results from multiple rules, we considered the Maximum function as an accumulation method that can be represented as follows:

(20) $$\mu_{target}=max\left[\mu_{localMEC}^{R1},\;\mu_{neighboringMEC}^{R2},\;\mu_{cloud}^{R3}\right]$$

After calculating the value of $\mu_{localMEC}^{R1}$, $\mu_{neighboringMEC}^{R2}$, and $\mu_{cloud}^{R3}$ from Equations (17)–(19), we can determine the value of the target server in the accumulation phase by using Equation (20), which is 0.2. Therefore, the target server is the neighboring edge server.

(21) $$\mu_{target}=max\left[0,\;0.2,\;0\right]\;=\;0.2$$

### 4.3. Defuzzification

**Figure 9:**
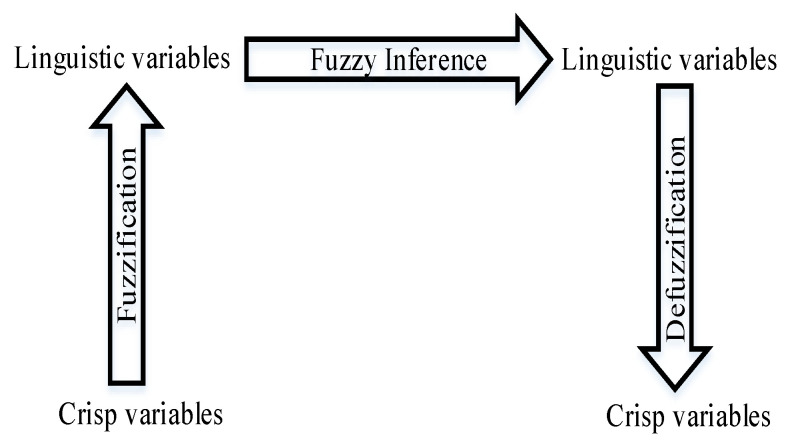
Fuzzification and defuzzification process.

Defuzzification is the process of converting into a crisp value the output of the aggregated fuzzy set produced by the inference mechanism. It is an inverse transformation, compared with the fuzzification process, which is shown in Figure 9.

The result of fuzzy inference is a linguistic value that translates into a numerical value in the defuzzification step. There are different methods for defuzzification, including fuzzy clustering defuzzification (FCD), weighted fuzzy mean (WFM), mean of maximum (MOM), and center of gravity (COG) [39]. The most popular and commonly used method is COG, which is the defuzzification step in our proposed system. This method determines the value of the center of gravity under the curve and returns the corresponding crisp value. After implementing the COG method in our proposed system, we obtained the crisp value, $x^{*}$, which is in the range [0, 100]. Based on the value of $x^{*}$, we defined the offloading decisions, all of which are shown in Table 4.

**Figure 10:**
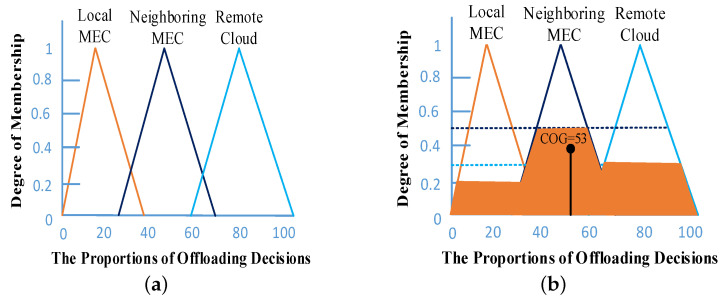
Defuzzification process: (**a**) output membership function; (**b**) the centroid defuzzification process.

The centroid defuzzification process is shown in Figure 10. For example, if the value of $\mu_{localMEC}$, $\mu_{neighboringMEC}$, and $\mu_{cloud}$ are calculated as 0.2, 0.5, and 0.3 respectively, then the crisp result after the centroid defuzzfication process will be 53, as shown in Figure 10b. So, based on the crisp result, the task is offloaded to the neighboring edge server. Algorithm 1 is the FTOM algorithm. Mathematically, the COG method is represented as follows.

(22) $$COG,\;x^{*}=\frac{\int x\mu (x)dx}{\int \mu (x)dx}$$

**Algorithm 1** Fuzzy Decision-Based Task Offloading Management (FTOM) Algorithm **Input:** The incoming task, T **Output:** Target offload node, O1:Read the network topology;2:Read the profile of incoming task T;3:$f_{v}$← FuzzyLogic(τ, ι, *d*, η, *w*); // Output value that fuzzy logic returns4:Calculate the center of gravity value for crisp output, COG ← Equation (22);5:Offloading decision, O ← Table 4;6:**return** O;

## 5. Performance Evaluation

In this section, we evaluate the effectiveness of our proposed FTOM scheme in terms of task failure rate, task processing latency, task completion time, and number of successfully executed tasks for different VM conditions in MEC-enabled networks with respect to various user devices through the EdgeCloudSim simulator [41]. To verify the performance, our proposed scheme was compared with five other benchmark task offloading schemes: local edge offloading (LEO), two-tier edge orchestration-based offloading (TTEO), fuzzy orchestration-based load balancing (FOLB), fuzzy workload orchestration-based task offloading (WOTO), and fuzzy edge-orchestration based collaborative task offloading (FCTO). In the LEO scheme, all users offload and execute their tasks by using the local MEC server. In the TTEO, FOLB, and WOTO schemes, all the neighboring edge servers and the remote cloud are connected to the orchestrator. The orchestrator distributes the incoming tasks and processes those tasks by using the edge servers and the cloud. On the other hand, orchestrator of the FCTO scheme distributes the incoming tasks among the edge servers. In order to present a realistic simulation for different real-life scenarios, we used three different applications during the experiments: an augmented reality (AR) application, an infotainment (I) application, and a health monitoring (HM) application [42,43,44]. Among them, the HM application is latency-sensitive, and the infotainment application is delay-tolerant. The AR application, however, is latency-sensitive as well as compute-intensive, requiring more CPU time. According to [36,38,41], Table 5 lists the key characteristic parameters of the AR, I, and HM applications, and the other simulation parameters used during the simulation are presented in Table 6.

Here, the tasks that are offloaded from the user device are represented as a set of predefined application categories, such as face recognition, infotainment services, and fall-risk detection. For example, in an AR application, a user wears smart glasses to upload images to the server for face identification. For a fall-risk detection service, the health monitoring application uses a foot-mounted inertial sensor that records the waking pattern of the user for a while; then, it sends the readings to a remote server for further processing. In Table 5, usages represent the percentage of mobile devices running for AR, I, and HM applications. In this study, we used 50%, 30%, and 20% for AR, I, and HM applications respectively. The task interarrival time depicts the frequency for transmitting the task to the orchestrator, which follows an exponential distribution. We considered the task interarrival time for AR, I, and HM applications were 2, 5, and 10 s respectively. We used a higher task interarrival time for HM application than others because we need to record the sensor data for a specific duration and send that collected data for further processing.

To identify the sensitivity of the task (delay-sensitive or delay-tolerant), we used the delay sensitivity value in our simulation. The offloaded task is considered delay-tolerant if the delay sensitivity value is low. Because the infotainment application is delay-tolerant, we used a delay sensitivity value of 0.3 during the experiment. On the other hand, the AR and HM applications are delay-sensitive, and thus, 0.9 and 0.7, respectively, were the delay sensitivity values. The task is generated during the active period but stays idle in the waiting period. For example, in the AR I, and HM applications, we use 40, 45, and 15 s for active mode and 20, 25, and 90 s for idle mode, respectively. In the AR and HM applications, a user uploads a large amount of data for service and receives a comparatively lower amount of data in response. Therefore, during the simulation, we considered upload and download data sizes of <1.5 MB, 25 KB> for the AR application and <1.25 MB, 250 KB> for the HM application. Moreover, with the infotainment application, a user sends a very small amount of data with a service request and the corresponding service returns a large amount of data in response. Thus, we used an upload data size of 25 KB and the corresponding downloaded service was 750 KB in response. The task length defines the needed CPU resources for the corresponding task in the giga instructions (GI) unit. In the simulation analysis, we used 50 mobile devices in the lightly loaded scenario and 500 mobile devices in the heavily loaded scenario. Moreover, we used 14 APs, and each AP was equipped with a single MEC server.

**Figure 11:**
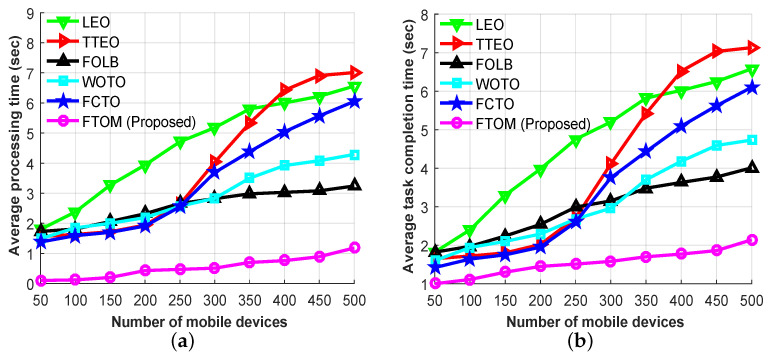
Performance evaluations based on all application types: (**a**) average processing times; (**b**) average task completion times.

To measure the efficiency of the proposed FTOM scheme, Figure 11a,b show the average processing time and the average task completion time (the *y*-axes), respectively, versus the number of mobile devices (the *x*-axes, varying from 50 to 500). From analyzing Figure 11a, the processing time tends to enhance in case of all scenarios to handle the excessive number of mobile devices, and the LEO scheme provides the worst performance than others. This is because the local MEC server experiences congestion due to its lower computing capabilities. On the other hand, the FOLB scheme provides better performance than the LEO scheme, since tasks are distributed between the MEC server and the remote cloud. Moreover, the FCTO scheme also provides better performance until 200 mobile devices than others except the FTOM scheme. In this scheme, tasks are easily distributed among the neighboring edge server. When it comes to the TTEO, WOTO, and our proposed FTOM scheme, they distribute the tasks among the MEC servers and the cloud. Therefore, for handling more mobile devices, the processing time does not increase, compared to the LEO scheme. However, when the number of mobile devices increases, for example, to 200, the average processing time for LEO, TTEO, FOLB, WOTO, FCTO, and our proposed FTOM scheme were 3.94, 1.94, 2.32, 2.19, 1.91, and 0.42 s, respectively. By comparing all schemes, the proposed FTOM scheme outperformed all the others as the load increased. In Figure 11b, the completion times for the above-mentioned task offloading schemes are given. The task completion time is derived by using the following formula: task completion time = processing time + network delay. Overall, the average task completion time tends to increase with increased numbers of mobile devices, and our proposed FTOM scheme showed the best performance, on average, because our proposed scheme can make dynamic decisions, and it efficiently balances both networking and edge computational resources, compared to the competitors. From the simulation results, we conclude that our proposed system can reduce the task completion time by approximately 66.6%, 61.5%, 47.9%, 49.8%, and 55% when compared to the LEO, TTEO, FOLB, WOTO, and FCTO schemes, respectively.

**Figure 12:**
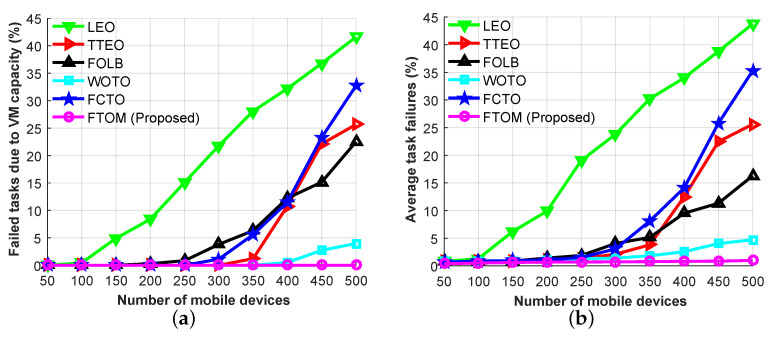
Performance analysis based on each application type: (**a**) failed tasks due to VM capacity; (**b**) average task failure rate.

Moreover, to verify the necessity of the proposed FTOM scheme, Figure 12a,b show another experiment to investigate the task failure rate in terms of different numbers of mobile devices. The task failure rate indicates the percentage of task failures out of the total number of tasks. Figure 12a shows the task failure rate based on VM capacity. During the simulation, we used four VMs for each MEC server. Figure 12a shows that the LEO scheme starts to experience congestion after 100 mobile devices, the FOLB and FCTO schemes starts getting congested after 250 and 300 mobile devices, respectively. Due to its limited computing capacity, the LEO scheme faces an overload problem after 100 mobile devices and starts to congest. The FOLB scheme distributes the tasks between the local MEC server with the cloud and the FCTO scheme distributes the tasks among the neighboring MEC server. Therefore, the FOLB and FCTO schemes can easily handle 250 and 300 mobile devices respectively without congestion. After that, due to the WAN delay, the FOLB scheme faces congestion and due to the overloaded problem, the FCTO scheme faces congestion. On the other hand, the other three offloading schemes distribute the tasks among MEC servers and the cloud. Thus, the TTEO and WOTO schemes start to experience congestion after 350 and 400 mobile devices, respectively, and our proposed FTOM scheme can handle 500 devices without congestion. This is because our proposed system can utilize local and neighboring MEC servers more efficiently than its competitors in a dynamic environment. Similarly, Figure 12b shows the average task failure rate for the aforementioned task offloading schemes. There are three main factors contributing to task failure: server capacity, network delay, and mobility. In these experiments, we considered those three factors when calculating the average task failure rate. Analyzing Figure 12b, the task failure rate is approximately zero until there are 100 mobile devices. However, the situation changes as the number of devices increases. A heavily loaded system increases the task failure rate in all scenarios due to congestion. For example, the task failure rate rapidly increased from 1.3% at 100 devices to 43.7% at 500 devices in the LEO scheme; from 3.8% at 350 devices to 25.6% at 500 devices in the TTEO scheme; from 4% at 300 devices to 16.3% at 500 devices in the FOLB scheme; from 2.6% at 400 devices to 4.7% at 500 devices in the WOTO scheme; from 3% at 300 devices to 35.3% at 500 devices in the FCTO scheme; and from 0.82% at 400 devices to 0.98% at 500 devices in our proposed FTOM scheme. Comparing all the schemes, our proposed FTOM provided a lower task failure rate than the others because it makes better decisions about sending tasks to MEC servers and, based on the network condition, sending some tasks to the remote cloud.

**Figure 13:**
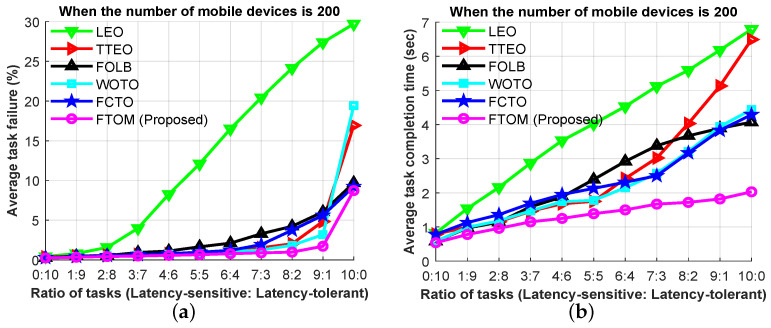
Performance analysis based on latency-sensitive to latency-tolerant task ratio: (**a**) average task failure rate; (**b**) average task completion time.

By varying the ratio between the latency-sensitive AR application and the latency-tolerant infotainment application, Figure 13a,b show the task failure rate and the task completion time, respectively, for the aforementioned task offloading schemes. In these experiments, we considered the average task length of the AR application to be higher than the infotainment application, because the AR application is not only latency-sensitive but also compute-intensive. Initially, we considered the ratio between two applications to be 0:10, meaning all the offloaded tasks are latency-tolerant. Then, the task failure rate of the LEO, TTEO, FOLB, WOTO, and FTOM schemes were 0.43%, 0.36%, 0.25%, 0.25%, and 0.23%, respectively. The task failure rate is low at this ratio because all the tasks are latency-tolerant. On the other hand, if we use all latency-sensitive applications, the ratio is 10:0. In this scenario, the task failure rate of the LEO, TTEO, FOLB, WOTO, FCTO, and FTOM schemes was 29.71%, 16.93%, 9.69%, 19.43%, 9.23%, and 8.73%, respectively. Therefore, it is seen that, when we use all latency-sensitive applications, the FCTO scheme provides lower task failure rate than others except the FTOM scheme. From the above analysis in Figure 13a, we observe that when there are more latency-sensitive tasks compared to latency-tolerant tasks, the average task failure rate increased in all scenarios. But our proposed FTOM scheme reduced the average task failure rate, compared to the others, because the proposed system utilizes local and neighboring MEC servers for offloading latency-sensitive tasks and, based on the network condition, utilizes a remote server to offload latency-tolerant tasks. Similarly, Figure 13b shows the task completion times for the different ratios between latency-sensitive and latency-tolerant applications. In this experiment, latency-sensitive AR applications are relatively heavy, compared to latency-tolerant applications. Thus, the average task completion time of the latency-sensitive tasks is higher than the latency-tolerant tasks. Our proposed scheme reduces the task completion time in all scenarios, compared to the other schemes.

**Figure 14:**
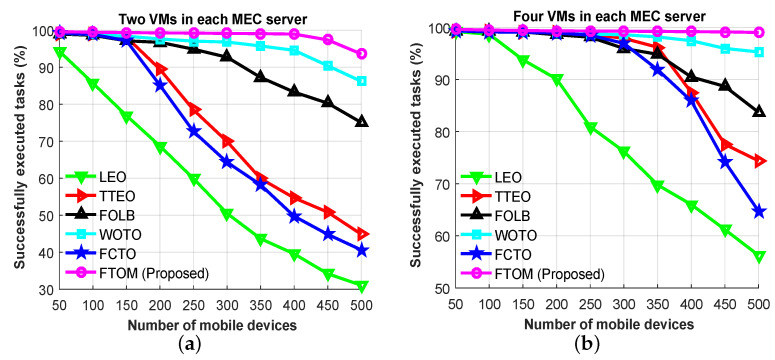
Successfully executed tasks versus the number of mobile devices: (**a**) with two VMs in each MEC server; (**b**) with four VMs in each MEC server.

Furthermore, Figure 14a,b, show the successfully executed offloaded tasks for two different MEC server capacities versus the number of mobile devices. From the simulation results, we observed that most of the offloaded tasks were executed successfully when the system was lightly loaded. However, this success rate decreased because of the growing number of devices. In Figure 14a,b, for two VMs and four VMs deployed, respectively, the number of successfully executed tasks dropped after 150 mobile devices had been added and after 250 mobile devices had been added for all schemes except LEO. At both capacities, the LEO scheme could not handle more tasks due to congestion in the VMs. Thus, with two VMs in each MEC server, the number of successfully executed tasks rapidly dropped from 94.3% at 50 devices to 31% at 500 devices. With four VMs in each MEC server, successfully completed tasks dropped from 99.2% at 50 devices to 56.2% at 500 devices under LEO. On the other hand, with two VMs in each MEC server, the number of successfully executed tasks dropped from 99% at 50 devices to 44.8% at 500 devices when using the TTEO scheme. For the FOLB scheme, completed tasks dropped from 98.8% at 50 devices to 75% at 500 devices, for the WOTO scheme, completed tasks dropped from 99.2% at 50 devices to 86.2% at 500 devices and for the FCTO scheme, completed tasks dropped from 99.15% at 50 devices to 40.6% at 500 devices. Our proposed FTOM scheme, however, saw the successful execution rate drop from 99.6% at 50 devices to 93.5% at 500 devices. Figure 14a,b, show that the rate of successfully executed tasks tends to increase with the increasing number of VM. When comparing the five schemes, our proposed FTOM approach outperformed the others because it can alleviate the load on the local edge server and efficiently distribute tasks to neighboring MEC servers and the remote cloud based on the network condition. After analyzing the simulation results, we can summarize that using our proposed FTOM scheme improves the successfully executed task rate by almost 68.5% compared with the LEO scheme, by 32.4% compared with the TTEO scheme, by 8.9% compared with FOLB, by 3.2% compared with WOTO, and by 38.6% compared with FCTO.

**Figure 15:**
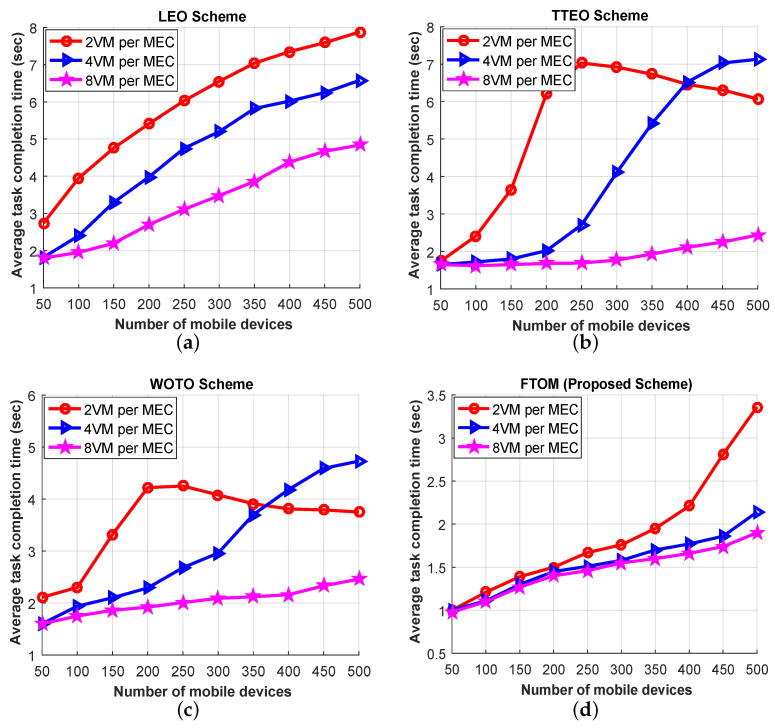
Performance evaluation based on each application types for different VM condition: (**a**) LEO scheme; (**b**) TTEO scheme; (**c**) WOTO scheme; (**d**) FTOM scheme.

Finally, in the last simulation result, the effect of different MEC server capacities in terms of the number of mobile devices was investigated, and the results are shown in Figure 15. In this experiment, we assigned three different numbers of VMs (eight, four, and two) to each MEC server. Figure 15 shows that the completion time with the LEO scheme was worse than the others in all scenarios. The main reason is that, for processing tasks on the local MEC server, many users wait a long time in the queue. For example, when the number of mobile devices is 100 and two VMs are deployed in each MEC server, the completion times for the LEO, TTEO, WOTO, and FTOM schemes were 3.95, 2.41, 2.3, and 1.21 s, respectively. However, the completion times for the LEO, TTEO, WOTO, and FTOM schemes were 1.95, 1.62, 1.75, and 1.1 s, respectively, when eight VMs were deployed in each MEC server. From the above analysis, we can say that each scheme can handle more user devices, as well as reduce the average task completion time, if the number of VMs is increased. However, our proposed FTOM scheme outperformed in all the scenarios, since it can avoid congestion and balances loads more efficiently among the MEC servers in the same tier.

## 6. Discussion

In this section, we have summarized the previously proposed various task offloading schemes in MEC-enabled networks and analyzed the performance evaluation results with respect to various evaluation metrics to show the effectiveness of our proposed FTOM scheme. The different task offloading schemes for various scenarios, including single/multiple users, single/multiple tasks, and different computing locations (local MEC/neighboring MEC/cloud server) are summarized in Table 7. The previous work mostly focused on vertical offloading between MEC and the cloud or on horizontal offloading among neighboring MEC servers. For example, Chen et al. [45] considered multi-user, single task, and local MEC computation offloading scheme. They ignored the neighboring MEC as well as the remote cloud server. Dinh et al. [46] considered single-user and multi-task offloading schemes. For processing the offloaded task, authors utilized local MEC as well as neighboring MEC servers. However, they ignored the remote cloud server which has powerful computing capabilities and did not consider the multi-user scenarios. On the other hand, Liu et al. [47] considered multi-user and single task offloading scheme. For task offloading, authors considered local MEC and remote cloud servers while they ignored neighboring MEC servers. Most of the previous work did not consider the collaborative integration between vertical and horizontal task offloading schemes. Therefore, to take the advantage of both task offloading schemes, in this paper, we propose an efficient fuzzy decision–based task offloading management (FTOM) scheme. Our proposed scheme used multi-user and multi-task offloading scenarios. Moreover, to utilize the neighboring MEC servers as well as a remote cloud server, our FTOM scheme considers vertical and horizontal task offloading schemes.

Table 8 is summarized from Figure 11, Figure 12, Figure 13, Figure 14 and Figure 15, which shows the comparisons of our scheme with the existing task offloading approaches. We have used many key performance evaluation metrics in this study to analyze the effectiveness of our proposed FTOM scheme. From Table 8, we observe that our proposed FTOM scheme provides lower processing and task completion time compared with other task offloading schemes. It reduces task completion time by approximately 66.6%, 61.5%, 47.9%, 49.8%, and 55% when compared to the LEO, TTEO, FOLB, WOTO, and FCTO schemes, respectively. Moreover, due to the VM capacity and overloaded problem, LEO, TTEO, FOLB, WOTO, and FCTO schemes starts getting congested after 100, 350, 250, 400, and 300 mobile devices respectively. On the other hand, our proposed scheme can handle 500 mobile devices without any congestion. Furthermore, the average task failure rate and task completion time increase in all scenarios when there are more latency-sensitive tasks compared to latency-tolerant tasks, and our proposed scheme outperforms others. When the system was lightly loaded, most of the offloaded tasks were executed successfully in all task offloading schemes. However, our proposed FTOM scheme improves the successfully executed task rates by almost 68.5%, 32.4%, 8.9%, 3.2%, and 38.6% compared with LEO, TTEO, FOLB, WOTO, and FCTO schemes respectively. Therefore, after analyzing Table 8, we can conclude that our proposed system significantly improves the rate of successfully executing offloaded tasks compared to others.

## 7. Conclusions

Efficient task offloading management in a MEC-enabled network is an intrinsically difficult online problem because the environment of edge computing is extremely dynamic, and the states of computing resources change rapidly based on the offload requests. On the other hand, without proper task offloading management, the distinct MEC server is not fully utilized or is sometimes overloaded by handling so many user requests. To handle this uncertainty and provide an automated management system, we proposed an efficient fuzzy decision-based task offloading management (FTOM) scheme. Our proposed approach makes dynamic decisions as to where to offload incoming tasks based on the states of server resources, the network conditions, and the latency sensitivity of the tasks. Moreover, our proposed system utilizes nearby MEC servers as well as the remote cloud to handle the overload problem and increase performance in a MEC server. To offload decisions, our system analyzes the computing resources to determine if they are already overloaded or underutilized. It can efficiently balance both networking and computational resources, where small and latency-sensitive tasks are better offloaded to a local or nearby MEC server. To evaluate our FTOM scheme, we used infotainment, augmented reality, and health monitoring applications and compared the proposed scheme with five benchmark schemes. According to the evaluations, our proposal outperformed its competitors in terms of task failure rate, task completion latency, and number of successfully executed tasks in all scenarios. For future work, we will consider a machine learning approach to efficient task offloading in MEC-enabled networks

## Figures and Tables

**Table 1:** Comparison between mobile cloud computing (MCC) and multi-access edge computing (MEC) computing architectures.

Technical Aspect	MCC	MEC
Deployment	Centralized	Dense and distributed
Architectural style	Client-server	Peer-to-peer
Computing capabilities	Higher	Lower
Network access	Multi-hop	Single-hop
Support for client mobility	Limited	Supported
Support for server mobility	Not supported	Supported
Number of nodes	Small (100–1000)	Large (billions)
Heterogeneity	Limited support	Full support
Latency	High	Very Low
Storage capacity	Ample	Limited
Location	Large data center	With network ingress
Hierarchy	2 tiers	3 tiers

**Table 2:** Fuzzification input variables [36,38].

Input Variables	Notation	Fuzzy Set	Range
Task size (GI)	τ	Small	0–8
		Medium	6–18
		Large	16–50
Local MEC VM utilization (%)	ι	Light	0–40
		Normal	30–70
		Heavy	60–100
Network delay (ms)	*d*	Low	0–4
		Medium	2–12
		High	10–100
Neighboring MEC VM utilization (%)	η	Light	0–40
		Normal	30–70
		Heavy	60–100
WAN bandwidth (Mbps)	*w*	Low	0–4
		Medium	3–7
		High	6–21

**Table 3:** Example fuzzy rules.

Rule Index	Task Size (τ)	Local MEC VM Utilization (ι)	Network Delay (*d*)	Neighboring MEC VM Utilization (η)	WAN Bandwidth (*w*)	Offload Decision
R1	Small	Light	High	Normal	Low	Local MEC Server
R2	Medium	Heavy	Low	Light	Medium	Neighboring MEC Server
R3	Medium	Heavy	Medium	Heavy	High	Remote Cloud
R4	Small	Heavy	Low	Normal	Low	Neighboring MEC Server
R5	Large	Low	High	Heavy	Low	Local MEC Server
R6	Small	Normal	Low	Light	Medium	Neighboring MEC Server
R7	Large	Heavy	Medium	Heavy	High	Remote Cloud

**Table 4:** Offloading Decisions.

Target Offloading Node	Range
Local MEC Server	0–40
Neighboring MEC Server	30–70
Remote Cloud Server	60–100

**Table 5:** Applications used in the simulations [36,41].

	Augmented Reality	Infotainment	Health Monitoring
	(AR)	(I)	(HM)
Usage (%)	50	30	20
Interarrival time of tasks (s)	2	5	10
Delay sensitivity (%)	0.9	0.3	0.7
Idle period (s)	20	25	90
Active period (s)	40	45	15
Upload data size (KB)	1500	25	1250
Download data size (KB)	25	750	250
Average task length (GI)	20	7.5	2.5
Task utilization of the VM (%)	10	5	2

**Table 6:** Simulation parameters [36,38,41].

Parameter	Value
Number of mobile devices	500
Number of edge servers	14
Number of VMs per edge server	2∼8
Number of VMs in the cloud	4
VM processing speed per edge server	10 GIPS
VM processing speed in the cloud	100 GIPS
WAN/WLAN bandwidth	Empirical
MAN bandwidth	MMPP/M/1 model

**Table 7:** Summary of different task offloading in MEC-enabled networks.

Publication	User		Task		Computing Location		Cloud Server
	Single	Multiple	Single	Multiple	Local MEC	Neighboring MEC	
Bi and Zhang [16]		✓	✓		✓		
Ning et al. [20]	✓	✓	✓		✓	✓	✓
Huang et al. [25]		✓		✓	✓		✓
Hossain et al. [27]		✓		✓	✓	✓	
Chen et al. [48]		✓		✓	✓	✓	
Huang et al. [49]		✓		✓	✓	✓	✓
Sonmez et al. [38]		✓		✓	✓	✓	✓
Li et al. [50]		✓	✓		✓	✓	
Chen et al. [45]		✓	✓		✓		
Dinh et al. [46]	✓			✓	✓	✓	
Liu et al. [47]		✓	✓		✓		✓
Wei et al. [51]	✓			✓	✓		
Our Work		✓		✓	✓	✓	✓

**Table 8:** Results summary of different methods.

Evaluations Metrics	Methods					
	LEO	TTEO	FOLB	WOTO	FCTO	FTOM
Average processing time (s)	4.58	3.91	2.57	2.87	3.38	0.53
Average task completion time (s)	4.61	4.01	2.96	3.08	3.43	1.54
Failed tasks due to VM capacity (%)	18.93	5.98	6.12	0.72	7.4	0
Average task failure (%)	20.79	7.12	5.21	1.94	9.12	0.70
Average completion time for different ratio of tasks (s)	3.92	2.63	2.41	2.19	2.28	1.35
Average task failure for different ratio of tasks (%)	13.2	2.75	2.76	2.73	2.3	1.44
Successfully executed tasks for 2VM MEC server (%)	58.43	74.4	90.47	95.46	71.04	98.49
Successfully executed tasks for 4VM MEC server (%)	79.21	92.88	94.78	98.06	90.85	99.29
